# Functional production of clostridial circularin A in *Lactococcus lactis* NZ9000 and mutational analysis of its aromatic and cationic residues

**DOI:** 10.3389/fmicb.2022.1026290

**Published:** 2022-11-23

**Authors:** Fangfang Liu, Auke J. van Heel, Jingqi Chen, Oscar P. Kuipers

**Affiliations:** Department of Molecular Genetics, Groningen Biomolecular Sciences and Biotechnology Institute, University of Groningen, Groningen, Netherlands

**Keywords:** RiPPs, circularin A, *Lactococcus lactis*, antimicrobial activity, biosynthesis, mutagenesis

## Abstract

Circular bacteriocins, also known as bacterial head-to-tail cyclized peptides, are a subgroup of ribosomally synthesized and post-translationally modified peptides (RiPPs). Compared with their conventional linear counterparts, circular bacteriocins are highly stable over a broad temperature and pH range, and circularization decreases proteolytic degradation by exopeptidases. These features render them great potential as scaffold candidates to withstand strident conditions in food- and pharmaceutical applications. However, the biosynthesis and bioactivity of circular bacteriocins still remain largely unknown. To investigate and gain more insights into the biosynthesis of circular bacteriocins and to achieve efficient production and characterization of bacteriocin variants, we developed an efficient cloning and heterologous expression system for clostridial circularin A and successfully produced this circular peptide in *Lactococcus lactis* NZ9000. We report three system formats with single plasmid or plasmid combinations to achieve successful cloning and functional production of circularin A in *L. lactis*. These systematic varieties enabled us to choose the appropriate method to efficiently obtain various constructs with desired properties. With the established heterologous systems in *L. lactis*, we performed several mutagenesis studies in the precursor peptide to study its structure/function relationships. The overlay activity assay revealed that these mutant variants had variable effects on different indicator strains: lysine substitution for certain glutamine residue(s) greatly decreased its bioactivity against *Clostridium perfringens* and *L. lactis* NZ9000, and alanine replacement for the cationic residues significantly reduced the activity against *Lactobacillus sake* ATCC 15521, whereas alanine substitution for the aromatic residues decreased its bioactivity against all three testing strains dramatically. Moreover, the conditions for bacteriocin production were optimized. Results show that supplementing the minimal medium with extra glucose (or sucrose) and immediate nisin-induction improved the peptide yield significantly. Briefly, we developed an excellent system for the production of circularin A and a wide range of variant peptides in a convenient host, as well as a method for fast detection of peptide production and activity. This system facilitated our mutagenesis studies which provided valuable insights into the effects of mutating specific residues on its biosynthesis and bioactivity, and will eventually enable more complex research into the biosynthesis of circularin A.

## Introduction

Ribosomally synthesized and post-translationally modified peptides (RiPPs) represent a diverse group of natural products, often containing particular structural elements (Arnison et al., 2013; Montalbán-López et al., 2021). Their structural diversity originates from the post-translational modification process, during which non-canonical amino acids (aa) or unique structural moieties can be formed, such as lanthionine or methyl-lanthionine in lanthipeptides, the specific knotted structure in lasso peptides, and head-to-tail ligation in circular bacteriocins. Because of the extensive post-translational modifications, RiPPs display various bioactivities, including potent antimicrobial properties (Arnison et al., 2013). Moreover, the ribosomal origin of RiPPs facilitates their bioengineering in development and optimization of these antimicrobials to create new-to-nature compounds. In recent years, with the assistance of the rapid development of gene sequencing and genome mining techniques, RiPPs have attracted unprecedented research interest (Perez et al., 2018; Montalbán-López et al., 2021). As the biosynthetic pathways of a growing number of RiPP sub-groups have been revealed in the past decades, rationally designing novel molecules with desired properties has created great engineering potential in the RiPP family (Hudson and Mitchell, 2018).

Among the various RiPP classes [for an overview see Arnison et al. (2013) and Montalbán-López et al. (2021)], bacterial head-to-tail cyclized peptides, which are also referred to as circular bacteriocins, form a group characterized by N- to C-terminal ligation of the peptide backbone. Compared with their conventional linear counterparts, circular bacteriocins are highly stable in a broad range of temperatures and pH values, and the lack of free termini in circular bacteriocins decreases their proteolytic degradation by potential exopeptidases (Borrero et al., 2017; Golneshin et al., 2020; Gor et al., 2020; Vezina et al., 2020). In addition, circular bacteriocins generally exhibit a broad antimicrobial spectrum, including activity against food-borne pathogens (Nakamura et al., 2013; Gabrielsen et al., 2014; Grande Burgos et al., 2014) and clinically relevant multi-drug resistant pathogens (van Heel et al., 2017). Although nisin, a lantibiotic peptide containing unique intramolecular lanthionine rings, has a long history of being used as a food preservative and currently remains one of the most widely used bacteriocins, its relatively poor stability at neutral and alkaline pH limits its application (Xin et al., 2020). Notably, most of the circular bacteriocins can withstand strident conditions of high temperature and extreme pH values, making them appealing scaffold candidates for both food- and pharmaceutical applications (Gabrielsen et al., 2014; Perez et al., 2018).

**FIGURE 1:**
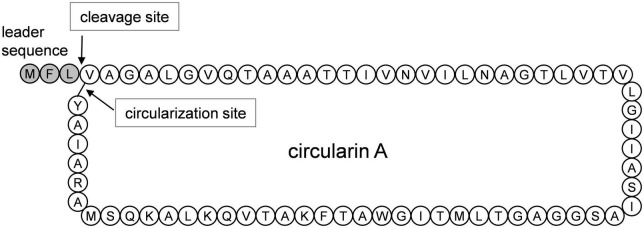
Circularin A precursor peptide with the putative maturation process. The peptide bond between Leu and Val is cleaved, and a new peptide bond is formed between Trp and Val as indicated by the arrows.

Despite the increasing interest, the biosynthetic mechanism of circular bacteriocins still remains poorly understood (Perez et al., 2018), which greatly limits the potential applications of circular bacteriocins as scaffold peptides to engineer new-to-nature compounds. Mutagenesis studies have been proven efficient in investigating the underlying mechanism. Up to now, extensive mutational analysis has only been performed for enterocin NKR-5-3B (Perez et al., 2017); site-directed mutagenesis has been performed for enterocin AS-48 (Sánchez-Hidalgo et al., 2008, 2010; Cebrián et al., 2010), and for plantacyclin B21AG (Gor et al., 2020). Expanding mutagenesis analyses to other known circular bacteriocins may help significantly in understanding the biosynthesis of circular bacteriocins (Perez et al., 2018). Circularin A, produced by *Clostridium beijerinckii* ATCC 25752, has been reported as a circular bacteriocin with a short leader sequence of 3 aa (Figure 1), and it has exhibited antimicrobial activity against various gram-positive bacteria, including all *Clostridium tyrobutyricum* strains tested (Kemperman et al., 2003b). Since bacteriocins are generally able to inhibit the growth of closely related bacteria, clostridial circularin A and its engineered derivatives have great potential to combat severe *Clostridium* infections, such as clinically pathogenic strains *Clostridium difficile* and *Clostridium perfringens*.

In this study, we first established three systemic formats with single plasmid or plasmid combinations to develop effective cloning strategies and achieve functional production of circularin A in *Lactococcus lactis* NZ9000. These systematic varieties enabled us to choose accordingly for efficiently cloning of various constructs with desired properties in an inducible expression system, facilitating future studies, for example, on biosynthesis and rationally engineered variants of circularin A. We then optimized the expression conditions for heterologous bacteriocin production. Additionally, residue substitution assays were performed with our newly established heterologous systems in *L. lactis*, to investigate the importance of certain residues on the biosynthesis and bioactivity of circularin A, which provided valuable insights into the effects of mutating specific residues on its biosynthesis and bioactivity.

## Materials and methods

### Bacterial strains, media, and reagents

The strains and plasmids used in this study and their references are summarized in Table 1.

**TABLE 1:** Bacterial strains and plasmids used in this study.

Strains or plasmids	Description^a^	References or sources
**Strains**		
*Clostridium beijerinckii* ATCC 25752	Circularin A producer, used for cloning the circularin gene cluster	NIZO
*Clostridium perfringens*	Bacteriocin indicator	NIZO
*Lactobacillus sake* ATCC 15521	Bacteriocin indicator	Lab collection
*Lactococcus lactis* NZ9000	Plasmid-free derivative of *L. lactis* MG1363, *pepN*:nisRK; used as heterologous production host and bacteriocin indicator	Kuipers et al., 1998
*Escherichia coli* DH5α	*sup*E44 Δ*lac*U169 (φ80 lacZΔM15) *hsd*R17 *rec*A1 *end*A1 *gyr*A96 *thi*-1 *rel*A1	Hanahan, 1983
**Plasmids**		
pMG36c	Cm*^r^*, pMG36e derivative with a constitutive promoter P_32_	van de Guchte et al., 1989
pNZ8048c	Cm*^r^*, carrying a nisin-inducible promoter P_nis_	Kuipers et al., 1998
pNZ8048e	Em*^r^*, pNZ8048c derivative	Lab collection
pTLR4	Em*^r^*/Amp*^r^*, carrying a nisin-inducible promoter, P_nis_	Lab collection
pTLR4-CirABCDE	Em^r^/Amp^r^, pTLR4 derivative containing *cirA, cirB, cirC, cirD, and cirE*	This study
pTLR4-CirΔA	Em^r^/Amp^r^, pTLR4-CirABCDE Δ*cirA*	This study
pNZ-CirA	Cm^r^, pNZ8048c containing *cirA*, also used for creating CirA mutants	This study
pNZ-CirB	Em^r^, pNZ8048e containing *cirB*	This study
pMG-CirΔB	Cmr, pMG36c containing *cirA, cirC, cirD, and cirE*	This study
pTLR4-CirΔB	Em^r^/Amp^r^, pTLR4-CirABCDE Δ*cirB*	This study
pNZ-CirA (W48A)	CirA peptide with point mutation of W48A	This study
pNZ-CirA (F51A)	CirA peptide with point mutation of F51A	This study
pNZ-CirA (K52A)	CirA peptide with point mutation of K52A	This study
pNZ-CirA (K57A)	CirA peptide with point mutation of K57A	This study
pNZ-CirA (K60A)	CirA peptide with point mutation of K60A	This study
pNZ-CirA (R65A)	CirA peptide with point mutation of R65A	This study
pNZ-CirA (Q56K)	CirA peptide with point mutation of Q56K	This study
pNZ-CirA (Q61K)	CirA peptide with point mutation of Q61K	This study
pNZ-CirA (Q56K/Q61K)	CirA peptide with point mutations of Q56K/Q61K	This study

^a^Cm^r^, chloramphenicol resistant; Em^r^, erythromycin resistant; Amp^r^, ampicillin resistant.

*Escherichia coli* DH5α was grown at 37°C in Luria–Bertani (LB) broth with vigorous agitation (220 rpm). *Lactococcus lactis* NZ9000 was grown at 30°C in GM17 broth containing 0.5% glucose (m/v). *Clostridium beijerinckii* ATCC 25752 and *C. perfringens* were grown anaerobically at 37°C in Reinforced Clostridial Medium (RCM). *Lactobacillus sake* ATCC 15521 was grown at 30°C in De Man, Rogosa, and Sharpe broth (MRS). Minimum Essential Medium (MEM) (Rink et al., 2005) was used whenever cell culture supernatant was subjected to peptide purification. For cell growth on plates, 1.5% agar was added to the corresponding broth. When required, ampicillin and chloramphenicol were used for *E. coli* DH5α at 100 μg/ml and 10 μg/ml, respectively. Chloramphenicol and erythromycin were used at 5 μg/ml each for *L. lactis* NZ9000. All the media and reagents mentioned above were purchased from Sigma-Aldrich unless stated otherwise.

### DNA amplification, plasmid construction, and transformation

The techniques of standard molecular cloning were performed as previously reported (Green et al., 2012). Genomic DNA from *C. beijerinckii* ATCC 25752 was extracted using the GenElute genomic DNA kit (Sigma-Aldrich, Zwijndrecht, Netherlands) and subjected to DNA amplification. The primers used for cloning were ordered from Biolegio (Nijmegen, Netherlands) (Supplementary Table 1). The NucleoSpin gel and PCR cleanup kit (BIOKÉ, Leiden, Netherlands) and the NucleoSpin Plasmid EasyPure kit (BIOKÉ, Leiden, Netherlands) were applied to purify PCR fragments and isolate plasmids, respectively. The obtained PCR products were ligated using Gibson Assembly Master Mix (BIOKÉ, Leiden, Netherlands) or USER Enzyme (NEB, Leiden, Netherlands) according to the manufacturers’ instructions. To achieve a high transformation efficiency, *E. coli* DH5α was used as an intermediate host for various constructs using the vector of pMG36c or pTLR4. The constructs using pNZ8048 vector were transformed directly into *L. lactis* NZ9000 competent cells by electroporation with the following settings: 2.25 kV, 25 μF, 200 Ω (Gene Pulser, Bio-Rad, Lunteren, Netherlands). The successful incorporation of desired mutations was verified by gene sequencing (Macrogen Europe, Amsterdam, Netherlands). The constructed *L. lactis* strains were stored at −80°C for further experiments.

### Expression, purification, and matrix-assisted laser desorption/ionization-time of flight (MALDI-TOF) spectrometry

For expression of circularin A, the heterologous host strain *L. lactis* was first inoculated at normal GM17 medium and grown overnight at 30°C. The next day, 2 ml fresh overnight culture was transferred into 100 ml of MEM supplemented with 0.5% glucose. The cells were grown at 30°C until the OD600 reached 0.5, then nisin was added at a final concentration of 5 ng/ml to induce bacteriocin production. After induction at 30°C for 16–20 h, the cell culture was centrifuged (8000 *g*, 15 min) and supernatant was collected for subsequent purification.

To purify circularin A, both methods of Trichloroacetic acid (TCA) precipitation and C18 purification were performed. The protocol for TCA precipitation was similar as described previously (Koontz, 2014). For C18 purification, 0.3 g C18 material (Sigma-Aldrich, Zwijndrecht, Netherlands) was packed in an open column and pre-washed with a solution consisting of 50% solution A (acetonitrile: isopropanol = 1:1, containing 0.2% TFA) and 50% solution B (milliQ water containing 0.2% TFA), then equilibrated with solution B. After column equilibration with 5 column volumes (CV), 100 ml collected supernatant was passed through the column by gravity, followed by a two-step wash and two-step elution process, which only differed in concentrations of solution A. The solutions applied to the washing steps were 20 and 50% solution A, respectively. The solutions used for the elution steps were 80 and 100% solution A, respectively. Each fraction was collected for 8 ml (around 10 column volume), freeze-dried (FreeZone, Labconco, Kansas City, MO, USA), then dissolved in 100 μl of 50% solution A, from which 1 μl sample was analyzed by matrix-assisted laser desorption/ionization-time of flight (MALDI-TOF) as previously described (Zhao et al., 2020). The rest of the peptide was stored at 4°C for further analysis such as protein gel and activity assay.

### Protein gel analysis

The production levels of the circularin A and its mutants were assessed by tricine-sodium dodecyl sulfate–polyacrylamide gel electrophoresis (tricine-SDS-PAGE) using a 16% running gel and a 4% stacking gel. The gels were prepared as described previously (Schägger, 2006). The amount of each peptide subjected to the gel was purified from 20 ml cell culture (1/5 of the purified peptide from the previous step). The C18 elution fractions were first freeze-dried and then dissolved in 20 μl of milliQ water. 5 μl of the loading buffer (10% SDS, 0.5% Bromophenol blue, 50% glycerol, 250 mM Tris–HCl, pH6.8) was added into the peptide sample, and the mixture was heated at 50°C for 30 min. 5 μl of the PageRuler Protein Ladder (Thermo Fisher, Groningen, Netherlands) was used as a protein molecular weight marker and run alongside the peptide samples. After separation, peptide bands were visualized by staining in 0.2% (m/v) solution of Coomassie Brilliant Blue R-250 (Bio-Rad, Merck, Darmstadt, Germany) for 20 min and subsequent destaining in a solution containing 10% acetate acid and 20% ethanol (v/v). After gel destaining, peptide yields were visualized and estimated by band intensities in Image Lab 3.0 (Bio-Rad Laboratories, Hercules, CA, USA).

### Optimization for peptide production

To achieve a better production of the antimicrobial peptide, various parameters were examined with a strain of *L. Lactis* containing the constructed plasmid pTLR4-Cir (Em*^r^*, erythromycin resistance). The expression and purification procedures of circularin A were similar as described above. Briefly, the “standard condition” prior to optimization was used as follows: the cells were grown in the MEM supplemented with 0.5% glucose, 1% glycerol, 5 μg/ml erythromycin; when the cell growth reached the stage of OD600 ∼ 0.5, 10 ng/ml nisin was added to the cell culture to induce bacteriocin production at 30°C for 16–20 h. The antimicrobial production under this condition was set as 100% and used for comparison to evaluate peptide production under conditions of the altered parameters. The optimized parameters were mainly studied on the effect of the modification of the growth medium, such as the amount of carbon source, the effect of glycerol and antibiotic. Moreover, the influence of nisin-induction at different cell growth phases was also investigated. Compared with the above “standard condition,” five expression conditions, each with one changed parameter, were implemented as follows: (1) nisin-induction from the start-point of the expression incubation; (2) additional 50 mm sucrose (∼1.7%, w/v) in growth medium; (3) additional 1.7% glucose in growth medium; (4) absence of erythromycin in growth medium; (5) absence of glycerol in growth medium. The effect of each parameter was investigated independently and the peptide production was evaluated with a scale of production level as 0–10% (±), 10–50% (+), 50–150% (++), 150–500% (+++). A negative control strain lacking the peptide precursor gene (Δ*cirA*) was also included in this experiment.

### Site-directed mutagenesis in *cirA*

To facilitate the investigation of circularin A mutants, site-directed mutagenesis was performed with the two-plasmid expression system (Cir-A +ΔA). In this system, primers were designed using the inverse PCR technique to insert desired mutations into *cirA*, while the rest of the circularin A gene cluster remained intact. The experimental procedures of DNA amplification, plasmid construction, and transformation were the same as described above. Finally, the correctly sequenced *cirA* mutants were transformed by electroporation into *L. lactis* NZ9000 harboring the rest of the biosynthetic proteins (*cirΔA*) as described earlier. All the primers used in this study are listed in Supplementary Table 1.

### Antimicrobial activity assay

Antimicrobial activity of strains was assessed by colony overlay assays. Specifically, GSM17 medium (containing 0.5% glucose and 0.5M sucrose) supplemented with 5 ng/ml nisin and 1.5% (w/v) agar, served as the first layer (bottom layer). 2 μl overnight culture producer strain was spotted on the bottom layer and incubated at 30°C for 4–6 h before the second layer (top layer) seeded with an indicator strain was poured on top. The indicator strains used in this study were *L. sake* ATCC 15521, *C. perfringens* and *L. lactis* NZ9000, respectively.

When *L. sake* ATCC 15521 was used as the indicator strain, it was firstly inoculated in MRS liquid medium and cultivated at 30°C for 2 days, then the obtained cell culture was diluted 1,000-fold into MRS medium supplemented with 1% agar (the second layer). When *L. lactis* NZ9000 was used as the indicator strain, it was firstly inoculated in GM17 liquid medium and grown overnight at 30°C, then the overnight cell culture was diluted 10,000-fold into GM17 medium supplemented with 1% agar (the second layer). When *C. perfringens* was used as the indicator strain, it was firstly grown anaerobically in RCM liquid medium at 37°C for 24–36 h, and then the obtained cell culture was diluted 1,000-fold into RCM supplemented with 1% agar (the second layer). Ultimately, these two-layer testing plates were incubated at 30°C for 36–48 h in the case of *L. sake* ATCC 15521 and 16–20 h in the case of *L. lactis* NZ9000. The *C. perfringens* two-layer testing plate was grown in an anaerobic chamber at 37°C for 24–36 h.

### Sequence alignment analysis and structural prediction

The aa sequence of circularin A (GenBank: AAN86036.1) was aligned with that of enterocin AS-48 (GenBank: AHL69645.1), the prototype of circular bacteriocins. The candidate sequences were obtained from the NCBI database and aligned using Clustal Omega.^1^ The alignment result was viewed and edited with Jalview version 2.11.2.1 (Geoff Barton’s group, University of Dundee).

The three-dimensional (3-D) peptide structure was modeled by input of the aa sequence of circularin A in the SWISS-MODEL website,^2^ then the PDB formatted file containing the structural information could be downloaded and edited in PyMol.^3^

## Results

### Functional production of circularin A in *Lactococcus lactis*

**FIGURE 2:**
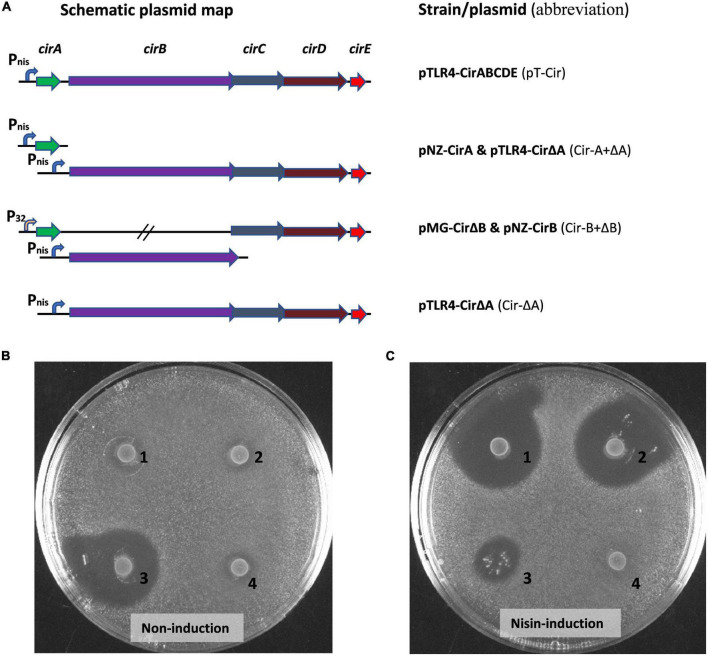
Heterologous production of circularin A in *Lactococcus lactis* NZ9000. **(A)** Construct design of the circularin A gene cluster in *Lactococcus lactis* NZ9000 and antimicrobial production of circularin A under various conditions: **(B)** non-induction, and **(C)** nisin-induction. (1) pTLR4-CirABCDE; (2) pNZ-CirA and pTLR4-CirΔA; (3) pMG-CirΔB and pNZ-CirB; (4) pTLR4-CirΔA. Their abbreviations are shown between brackets. Both the pNZ plasmid and the pTLR plasmid contain a nisin inducible promoter P_nis_, while pMG plasmid contains a constitutive promoter P_32_. The indicator strain: *Lactobacillus sake* ATCC 15521.

To achieve functional production of circularin A in *L. lactis*, various approaches were applied (Figure 2A): (1) *cirB* was separately put under the nisin-inducible promoter in the pNZ8048e plasmid, while the rest of the genes in the *cirABCDE* gene cluster were placed in pMG36c behind a constitutive promoter p32; (2) all five genes in the *cirABCDE* gene cluster were cloned into one single plasmid behind the nisin inducible promoter, creating pTLR4-CirABCDE, as well as (3) a variant form of the two-plasmid system (pNZ-CirA and pTLR4-CirΔA) was constructed to facilitate the later investigation of circularin A variants. Eventually, three different formats for cloning the *cirABCDE* gene cluster into *L. lactis* were completed, which included either one-plasmid approach pTLR4-CirABCDE, or the complementary constructs pNZ-CirA and pTLR4-CirΔA, and pMG-CirΔB and pNZ-CirB. The complementary design facilitates further research, especially on mutagenesis studies by creating an efficient cloning and production system to obtain sufficient amounts of various modified mutant peptides. It has been suggested that *cirB* is toxic to *L. lactis*, and that the circularin gene cluster expression under a constitutive promoter P_32_ was not possible to achieve (Kemperman et al., 2003a). Therefore, we firstly decided to separately place the *cirB* gene under a nisin inducible promoter, together with pMG-CirΔB to achieve heterologous expression of circularin A in *L. lactis*. Later, we noticed that pMG transformants were generally less efficiently transformed in *L. lactis* and inclined to grow much more slowly, which would greatly impede later studies on, for example, circularin mutagenesis. To create an efficient working system for heterologous and functional expression of circularin A in *L. lactis*, plasmids with a nisin-inducible promotor such as pNZ8048c and pTLR4 were selected and used for gene constructions. After multiple attempts, the whole *cirABCDE* gene cluster was successfully inserted into a single plasmid pTLR4 *via E. coli* DH 5α as an intermediate host. Meanwhile, another constructed strain with the complementary design of pNZ-CirA and pTLR4-CirΔA was also achieved. Overall, *L. lactis* NZ9000 strains with different constructs of the circularin A gene cluster, such as pT-Cir and its variant form Cir-A +ΔA, as well as Cir-B +ΔB, were made ready for further experiments.

To assess the functionality of our different expression constructs, we performed antimicrobial assays under both non-induction and nisin-induction conditions (Figures 2B,C). Without nisin-induction, the only one that showed antimicrobial activity was the host strain with the bacteriocin gene *cirA* placed behind a constitutive promoter, indicating undetectable leakage of the nisin promoter in the other constructs. When induced with nisin, all three constructed strains containing the whole *cirABCDE* gene cluster displayed antimicrobial activity, except the control strain which lacks the structural gene *cirA*. Notably, the strain with *cirB* separately situated behind the P_*nis*_ promoter in pNZ8048c had a growth issue with a clearly altered strain morphology from other strains under the nisin-induction condition, which is likely due to the toxicity of CirB overproduction.

**FIGURE 3:**
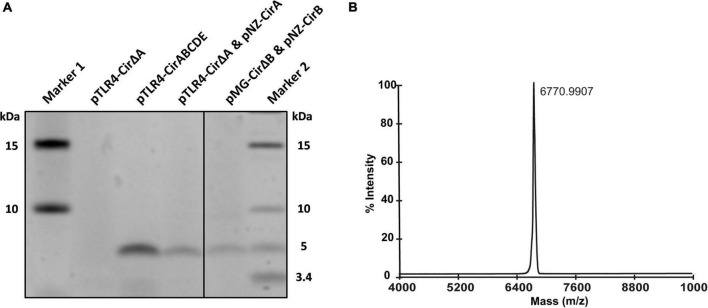
Production comparison and MS confirmation of circularin A. **(A)** Tricine-SDS-PAGE protein gel for antimicrobial production comparation in three expression constructs. **(B)** Matrix-assisted laser desorption/ionization-time of flight (MALDI-TOF) spectrum for molecular mass detection to confirm the production and modification of circularin A (theoretical mass: 6771.05 Da).

As shown by the antimicrobial tests, the production of the antimicrobial compound was achieved with various expression systems in *L. lactis*. For further confirmation, peptide expression and purification were carried out to assess the production yield and the modification level. Instead of complicated sequential steps of previously described purification methods that were commonly chosen for purification of circular bacteriocins, we applied a single-step purification by using a C18 open column. The obtained peptides from different eluent fractions were collected and subjected to an sodium dodecyl sulfate–polyacrylamide gel electrophoresis (SDS-PAGE) gel and MALDI-TOF analyses. The protein gel revealed a clear band from the 80% solvent elution fraction with a size close to 5K Dalton (Da) for all three expression constructs (Figure 3A). To determine the molecular size of this band, MALDI-TOF was performed and results showed a predominant peak with a detected mass of 6770.99 Da (Figure 3B). The theoretical mass of mature circularin A is 6771.05 Da, which is calculated based on the leader cleaved-off from the precursor peptide and the subsequent head-to-tail ligation. These results suggest that circularin A is fully modified in our heterologous production systems in *L. lactis* and that mature circularin A is eluted in fractions containing a high concentration of organic solvent (80%), which is consistent with its high hydrophobicity (GRAVY index of 1.007). Moreover, purification by TCA precipitation was also performed, and the corresponding mass of mature circularin A could be detected by MALDI-TOF, but with lower detection signal (data not shown).

### Extra sugar and early induction promote peptide production yield

Even though we successfully purified circularin A from all three expression systems in *L. lactis*, the production level was relatively low with our initial expression and purification conditions. To facilitate future mutagenesis studies that may significantly reduce peptide yields, we optimized the expression conditions to improve the antimicrobial production. The optimization for circularin A production mainly focused on the modifications of the growth medium, and the effect of each examined parameter was investigated independently. Compared with our standard condition, supplementation of the growth medium with additional amounts of sugar (e.g., 50 mM sucrose or 1.7% glucose) significantly increased the peptide production (Table 2 and Supplementary Figure 1). Moreover, nisin-induction from the start-point of the expression incubation also improved the peptide yield dramatically. Erythromycin, which was added to prevent the host from losing the target plasmid, had very little effect on the peptide production, and absence of glycerol in the growth medium decreased the peptide yield slightly. As expected, the control strain (Δ*cirA*) had no detectable antimicrobial peptide production. Based on the above observations, 2.2% glucose and immediate nisin-induction were applied to the expression conditions for subsequent peptide purification in this study. The production improvement of our two-plasmid constructed strain (Cir-A +ΔA) after optimization is shown in Supplementary Figure 2.

**TABLE 2:** The influence of various conditions on peptide production of circularin A with the constructed stain (pTLR4-CirABCDE).

Parameters	Peptide production^a^
**Standard conditions:**	
(1) Growth medium: Minimum Essential Medium (MEM) supplemented with 0.5% glucose, 1% glycerol, 5 μg/ml erythromycin; (2) Induced with 10 ng/ml nisin at the growth stage of OD600 ∼ 0.5.	++
**Changed parameters:**	
Nisin-induction from the beginning	+++
0.5% glucose + 50 mM sucrose (∼1.7%, w/v) in growth medium	+++
0.5% (+1.7%) glucose in growth medium	+++
No erythromycin in growth medium	++
No glycerol in growth medium	+

^a^Circularin A production under standard conditions is set as 100% for comparison. The relative peptide yield for each altered parameter is estimated based on the amount of peptide obtained from peptide purification, and is shown on a scale of 0–10% (±), 10–50% (+), 50–150% (++), 150–500% (+++).

#### Circularin A structural modeling and site-directed mutagenesis

**FIGURE 4:**
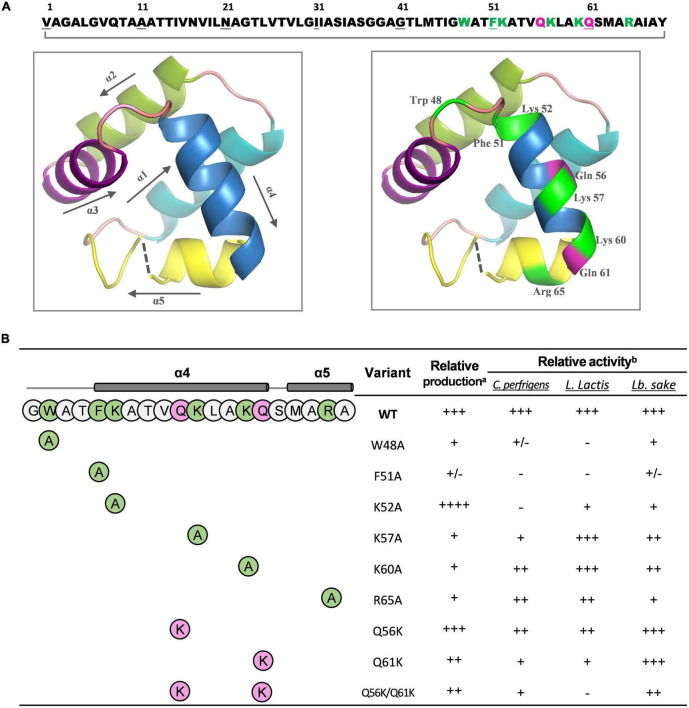
The predicted structure and the mutagenensis study of circularin A. **(A)** The amino acid (aa) sequence and the predicted three-dimensional (3-D) structure of mature circularin A. The N- and C-termini are ligated between Val1 and Tyr69 to form the mature circular peptide. The aromatic and cationic residues are indicated in green and the glutamine residues subjected to mutagenesis are shown in magenta. The 3-D structure of circularin A with the layout of each α-helix and their orientations is shown on the left, and the mutagenesis location sites highlighted are shown on the right. **(B)** The structure/production/bioactivity relationships of circularin A and its variants against three different indicator strains: *Clostridium perfringens*, *Lactococcus lactis* NZ9000, and *Lactobacillus sake* ATCC 15521. The production and bioactivity of the wild-type (WT) circularin A were set as 100% (+++) and used for comparison. The relative production and bioactivity of its variants were evaluated with a scale of 0–2% (–), 2–20% (+/-), 20–50% (+), 50–80% (++), 80–100% (+++), >100% (++++). ^a^Relative production of the variants was estimated by band intensities in protein gel. ^b^Relative activity of the variants was calculated by inhibition area in the colony overlay assay.

Bacteriocin circularin A, encoded by the *cirA* gene, is initially translated as a precursor peptide of 72 aa with a 3-aa leader peptide, and its aromatic and cationic residues are clustered at the C-terminus (Figure 4A). Based on the SWISS-MODEL analysis, circularin A has a protein sequence identity of 30.43, 25.40, and 25.45% with the structurally known AS-48 (Samyn et al., 1994), NKR-5-3B (Himeno et al., 2015), and carnocyclin A (Martin-Visscher et al., 2008), respectively. The predicted structure of circularin A consists of 5 α-helices and the circularization point is located within the α5-helix (Figure 4A). The layout of each α-helix and the residues subjected to respective site-directed mutagenesis are demonstrated in Figure 4A (left and right, respectively). Site-directed mutagenesis of circularin A in this study was focused on the importance of the cationic and aromatic residues on the biosynthesis and the activity of circularin A. To achieve that, we performed alanine substitution to the cationic and aromatic residues. In addition, we also investigated the effect of increasing the net charge of circularin A by performing lysine substitution of certain glutamine residue(s), since many antimicrobial peptides are overall strongly cationic. Site-directed lysine substitutions of Q56 and Q61 were selected based on the sequence alignment of circularin A and enterocin AS-48 (Supplementary Figure 3). The positions Q56 and Q61, but not Q8, are in line with the lysine residues of AS-48, and glutamine has a similar size as lysine, thus we introduce the positive charge(s) in circularin A with the choice of lysine substitutions at Q56 and Q61 positions. In total, 9 mutants were made, including W48A, F51A, K52A, K57A, R65A, Q56K, Q61K, and Q56K/Q61K.

Activity results show that these mutant variants have varying effects on different indicator strains (Figure 4B and Supplementary Figure 4A). Specifically, substituting aromatic residue (W48 or F51) with alanine lead to a nearly complete loss of antimicrobial activity against *C. perfringens* and *L. lactis* NZ9000, while significantly decreased its activity against *L. sake* ATCC 15521; replacing cationic residue (K52, K57, K60, or R65) with alanine showed varying effects: among these four mutants, K52A showed the most reduced antimicrobial activity against *C. perfringens* and *L. lactis* NZ9000, and the lowest activity level against *L. sake* ATCC 15521 was found for mutant R65A, followed by mutant K52A; introducing additional lysine to replace certain glutamine (mutants Q56K, Q61K, and Q56K/Q61K) showed an activity decrease against all three indicator strains with the highest activity found in mutant Q56K and the lowest activity found in mutant Q56K/Q61K.

**FIGURE 5:**
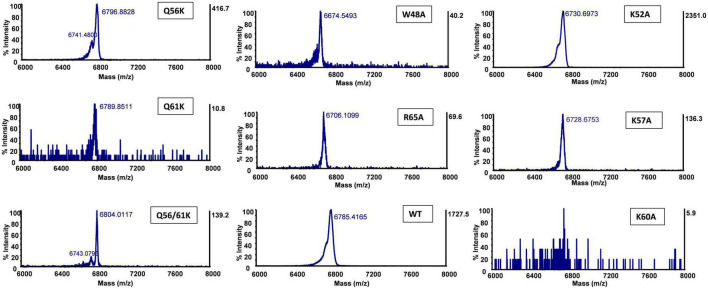
Matrix-assisted laser desorption/ionization-time of flight (MALDI-TOF) spectra of circularin A and variants. No calibration sample for MALDI-TOF detection was performed in this assay. The detected mass values are slightly different from their theoretical masses. So we evaluate the production of mutant peptides by comparing the mass reduction between the wild-type (WT) circularin A and the variants in Table 3.

To investigate the effect of each mutant on peptide production, peptide expression and purification were performed based on our optimized conditions. The production yield was estimated by comparing the thickness of peptide bands from a protein gel, assisted with MALDI detection to confirm the mass. Compared with wild-type (WT) bacteriocin, the production level significantly decreased for most alanine substitutions except for K52A, which showed a much thicker band (Supplementary Figure 4B). The fact that mutant K52A was highly produced with little observed antimicrobial activity against all three indicator strains suggested that the lysine at position 52 is critical for the activity of circularin A, and its substitution with alanine dramatically decreased antimicrobial activity but increased peptide yield. For lysine substitution (Q56K, Q61K, and Q56K/Q61K), a decent production level was witnessed from the protein gel, especially for mutant Q56K which also showed the highest activity among these three mutants. Notably, mutant Q56K/Q61K migrated slightly behind Q56K, Q61K, and WT bacteriocin in the protein gel, and its MALDI spectrum (Table 3) also revealed a mass approximately 21 Daltons greater than its theoretical mass, which might be due to the oxidation of a Met (or Trp) residue as reported in AS-48 (Cebrián et al., 2010). Mutant F51A and Mutant K60A were below the MALDI-TOF detection limit. The detailed spectra of circularin A variants are shown in Figure 5.

**TABLE 3:** Matrix-assisted laser desorption/ionization-time of flight (MALDI-TOF) mass detection to confirm the production of circularin A variants.

No.	CirA mutants	Theoretical mass decrease^a^	Observed mass decrease^b^	ΔM (Theoretical-Observed)^c^
1	W48A	115	111	+4
2	F51A	76	–	N/A
3	K52A	57	55	+2
4	K57A	57	57	0
5	K60A	57	–	N/A
6	R65A	85	79	+6
7	Q56K	1	−11	+12
8	Q61K	1	−4	+5
9	Q56K/Q61K	2	−19	+21

^a^The theoretical mass difference between the original amino acid (aa) (wild type) and the mutated amino acid (aa) (mutants).

^b^The detected mass difference of observed MALDI-TOF spectra between the wild-type (WT) circularin A and the mutant peptides. –, not identified.

^c^The mass difference between theoretical and observed values (ΔM), indicating the likelihood of fully modified circularin A variants. N/A, not applicable.

## Discussion

In this study we describe the expression systems we developed to achieve convenient cloning and functional production of circularin A in *L. lactis* NZ9000. Previously, heterologous expression of circularin A was reported in *Enterococcus faecalis* but with unstable bacteriocin production (Kemperman et al., 2003a). With our newly established expression systems in *L. lactis*, robust antimicrobial production of circularin A was achieved. Moreover, *L. lactis*, unlike *E. faecalis*, is a commonly chosen model organism (Kunji et al., 2003) in both laboratory research and industrial applications for various reasons such as easy manipulation and well-known safety. Based on our constructed expression systems, mutagenesis studies using the two-plasmid system (Cir-A + ΔA) proved to be quite efficient and successful, although various challenges have been reported previously at different stages for heterologous production of circular bacteriocins, such as the difficulty of obtaining positive clones (Kemperman et al., 2003a; Gor et al., 2020) at the molecular cloning level, and the difficulty in achieving antimicrobial production from hosts of different genera at the peptide expression level (Fernández et al., 2007; Kawai et al., 2009).

The overlay antimicrobial activity assay allowed for a quick screening of the activity for circularin A and its derivatives. Interestingly, the antimicrobial production of our constructed strain (pMG-CirΔB and pNZ-CirB) was clearly witnessed even under non-induction condition, whereas overexpression of CirB protein under nisin-induction condition caused stressful growth to the heterologous host (Figures 2B,C). To investigate if CirB is essential in circularin A biosynthesis, *cirB* knock-out strains (pMG-CirΔB and pTLR4-CirΔB) were constructed and assayed for overlay activity test, and neither strain showed antimicrobial activity (data not shown). These results indicated that the nisin promoter (P_*nis*_) in pNZ plasmid is not extremely tight and little production of CirB could fulfill its function in circularin A maturation. However, overexpression of *cirB* gene in a high-copy plasmid is harmful to the host *L. lactis* since an altered colony morphology was observed in the nisin induced strain (pMG-CirΔB and pNZ-CirB). A similar phenomenon was reported upon overproduction of the human KDEL receptor in *L. lactis*, as colony-counts assay showed that only 1 in 10^9^ colony-forming units survived induction when plated onto nisin-containing M17 plates (Kunji et al., 2003). Moreover, CirB is bioinformatically predicted to be a membrane protein which contains 11 putative transmembrane (TM) sequences (Kemperman et al., 2003a). Transmembrane peptides could be toxic to their bacterium host, which is likely caused by membrane destabilization (Montigny et al., 2004). Previous studies suggested that overproduction of membrane proteins in *L. lactis* could lead to growth stress (Kunji et al., 2003; Marreddy et al., 2011; Steen et al., 2011; Schlegel et al., 2014), and a growth study showed that overexpression of membrane proteins (YidC, YedZ, and LepI) in *E. coli* also severely reduced cell growth, with cell densities less than half that of the control strain (Wagner et al., 2007).

Another characteristic advantage of our methodology is the one-step purification procedure, which enables fast and efficient identification of peptide production when combined with mass spectrometry. As a circular bacteriocin, the mature circularin A is a secreted peptide, which facilitates purification from cell-free culture supernatant. Its high hydrophobicity also enables a distinct C18 purification profile from most other proteinaceous substances in the supernatant from a MEM culture. Although the commonly chosen methods for purification of circular bacteriocins make sequential use of various purification techniques (Sawa et al., 2009; Arakawa et al., 2010; Masuda et al., 2011; Al-Madboly et al., 2020), our spectrometry results suggest that the one-step purification method, together with the use of minimal medium, is sufficient to obtain the antimicrobial peptide (Figure 3). Subsequently, optimized conditions for bacteriocin production were implemented. We found that supplementing the growth medium with extra sugar (e.g., 50 mm sucrose or 1.7% glucose), as well as immediate nisin-induction, significantly increased the peptide yield. Data of growth curve showed that the ultimate cell density is proportional with increased amounts of sugar at a glucose concentration below 0.5% (m/v), and remains at a high level at glucose concentrations of 0.8–3.2%, while the ultimate cell density decreased significantly at a glucose concentration above 6.4% (Supplementary Figure 5). A previous report also suggested that increasing the concentration of the nutrients such as carbohydrate, aa and vitamin increased ultimate cell density (Kunji et al., 2003). Nisin-induction at the start-point lead to slower growth of the host strain in the early growth phase. It has been shown that high growth rates come with the cost of the expression level of metabolic enzymes and/or stress proteins (Molenaar et al., 2009; Scott et al., 2010; Nugroho et al., 2021).

The cationic and aromatic residues are highly conserved among putative and experimentally confirmed circular bacteriocins (Vezina et al., 2020), and have been demonstrated to play important roles in antimicrobial activity of plantacyclin B21AG (Gor et al., 2020). In terms of the mechanism of the antibacterial activity, it has been reported previously that the aromatic residues (such as Trp) have a specific affinity for the region close to the lipid carbonyl and promote membrane permeation, while the cationic residues (such as Lys) have the affinity to the negative charged lipid phosphate region and are believed to interact with negatively charged cell membrane (Braun and von Heijne, 1999; de Planque et al., 1999; Killian and von Heijne, 2000; Jiménez et al., 2005). Based on our predicted circularin A structure, 3 out of 4 cationic residues (Lys52, Lys57, and Lys60) and 1 out of 2 aromatic residues (Phe51) are located within the α4 helix with the other two (Trp48 and Arg65) in the flanking regions: Trp48 is situated at the loop region between the α3 and α4 helix, while Arg65 is located in the α5 helix. Antimicrobial activity overlay assay suggested that alanine substitution of the aromatic residues, especially mutant F51A, showed a severely decreased activity against all three indicator strains (Supplementary Figure 4A and Supplementary Table 2). The production of mutant F51A was barely seen in the protein gel, which indicated mutant F51A greatly hampered the peptide stability probably by compromised circularization (Gor et al., 2020). This is likely due to the fact that Phe51 is the first residue of the α4-helix, and mutation F51A alters the delicate secondary structure of the resulting peptide, causing inefficient biosynthesis. As a result, the F51A precursor may reside longer in the cell and easily get degraded, ultimately resulting in a lower yield. Site-directed substitution in enterocin AS-48 has shown that Trp24 is essential in the biological activity, and lysine substitutions for Gly13 and Leu40 show little effect on antimicrobial activity (Sánchez-Hidalgo et al., 2010). We observed that Trp48 is also important in the bioactivity of circularin A, and the introduction of lysine to replace certain glutamine residue(s) decreased antimicrobial activity, especially mutants Q61K and Q56K/Q61K. Interestingly, the alanine substitution for Lys52 improved peptide yield to 300–500%, but the peptide had little activity against all three indicator strains, indicating the important role of lysine at this position for bioactivity. Overall, alanine substitutions of the aromatic residues and cationic residues generally decreased production yield (except for K52A), resulting in a lower activity compared with the WT. The effects of decreased activity level also varied among these mutants. Compared with the α4-helical N-terminal residues (F51 and K52), the mutations of K57A and K60A retained better activity against all three tested indicator strains. Similarly, compared with the α4-helical C-terminal residues (Q61K), the mutant Q56K had a better antimicrobial activity and a higher production yield. This indicates that the residues at both ends of the α4-helix have a greater impact on antimicrobial production than residues in the middle.

In summary, we have genetically constructed a functional and efficient production system of circularin A in *L. lactis*. Additionally we have set up efficient methods to purify circularin A and its variants, and assess their antimicrobial activities and modifications. With these methods we enable more detailed investigation into the biosynthesis and bioactivities of bacterial head-to-tail ligated peptides. This will facilitate further studies and provide more insights into the underlying fundamental research such as the role of the leader peptide and the role of each biosynthetic protein in the circularization process.

## Data availability statement

The original contributions presented in this study are included in the article/Supplementary material, further inquiries can be directed to the corresponding author.

## Author contributions

FL, AvH, and OK discussed, conceived, and analyzed the study and corrected the manuscript. JC carried out some experimental work on plasmid construction. FL performed the experiments, analyzed the data, and drafted the manuscript. All authors read and approved the final manuscript.

## Footnotes

1
https://www.ebi.ac.uk/Tools/msa/clustalo/

2
https://swissmodel.expasy.org/interactive

3
https://pymol.org/2/
