# Supplementary material for: Functional production of clostridial circularin A in Lactococcus lactis NZ9000 and mutational analysis of its aromatic and cationic residues
Source: Front Microbiol. 2022 Nov 23;13:1026290. doi: 10.3389/fmicb.2022.1026290 (PMC9726714; doi:10.3389/fmicb.2022.1026290)
Supplement: Supplementary file 1 [file Data_Sheet_1.PDF]

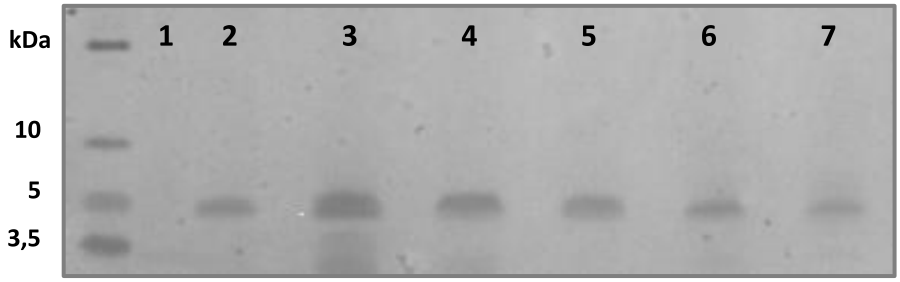

Supplementary Figure 1. Optimization of circularin A production with the construct pTLR4-CirABCDE in *L. lactis*. The examined parameters are investigated independently as follows: 1, negative control strain (Δ*cirA*); 2, antimicrobial production with "standard condition" for comparation; 3, nisin induction from the beginning; 4, 2.2% (w/v) glucose in growth medium; 5, 0.5% glucose + 1.7% sucrose (w/v) in growth medium; 6, no erythromycin in growth medium; 7, no glycerol in growth medium

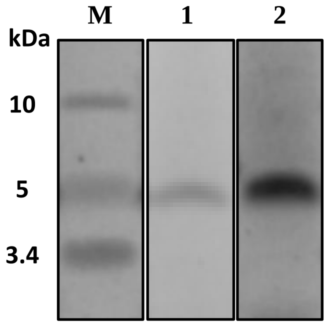

Supplementary Figure 2. Comparison of the production yields before and after optimization with our two-plasmid constructed strain (Cir-A+ΔA). M, protein marker; 1, peptide yield with the initial expression conditions of 0.5% glucose in growth medium and nisin-induction at the growth stage of OD600 around 0.5; 2, peptide yield with the optimized expression conditions of 2.2% glucose in growth medium and early nisin-induction at the start of the expression incubation.

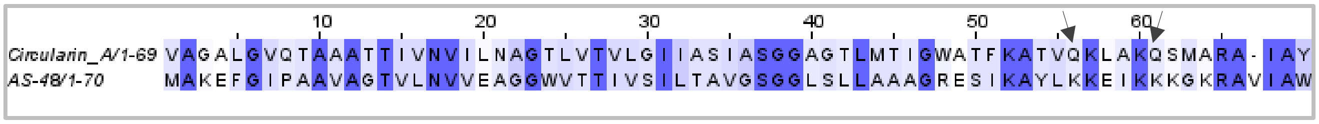

Supplementary Figure 3. Amino acid sequence alignment of circularin A with enterocin AS-48 was performed using Clustal Omega with default settings. The shading colour was shown in Jalview by setting conservation with percentage identity above 30%. The glutamine residues that were subjected to lysine substitution are indicated with black arrow.

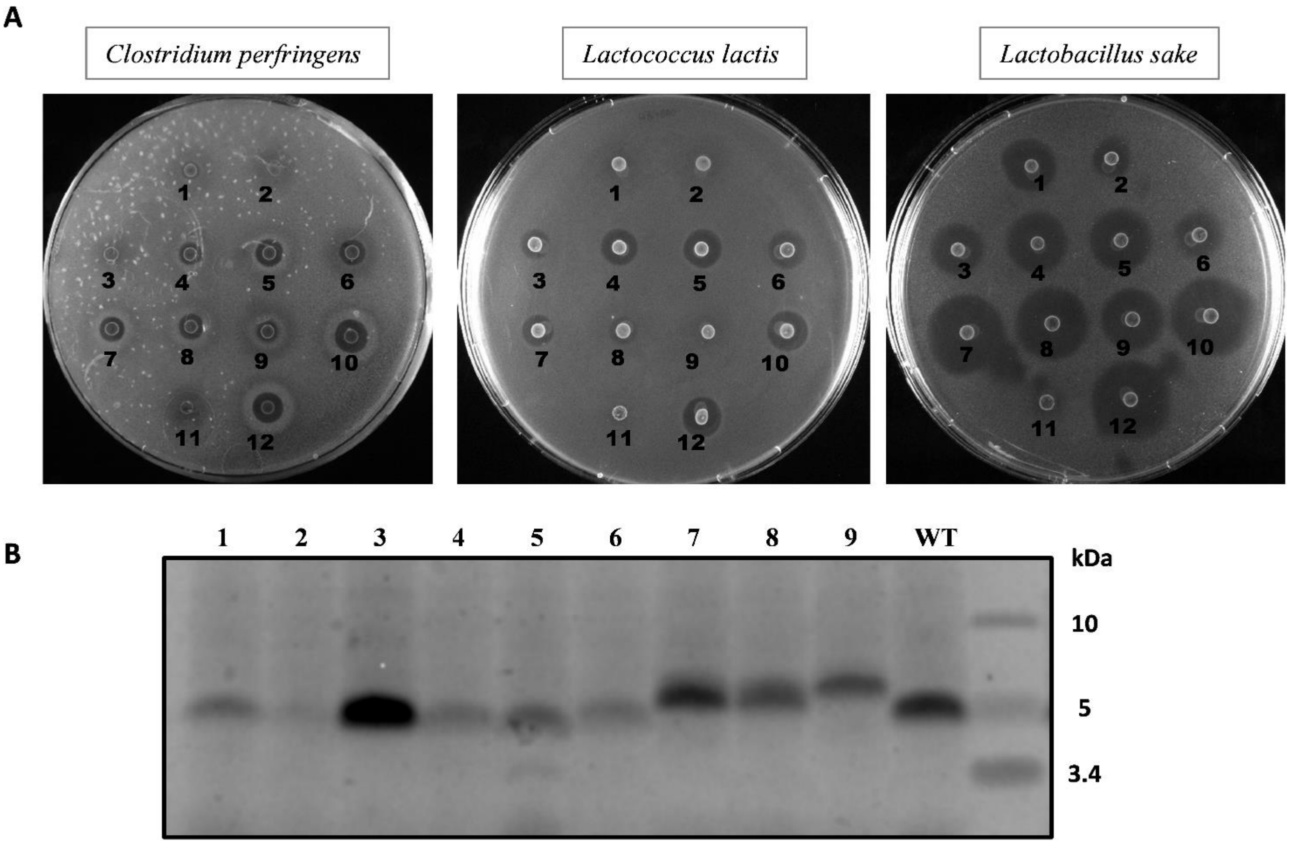

Supplementary Figure 4. **(A)** Overlay antimicrobial activity assay of circularin A and its variants against three different indicator strains: *Clostridium perfringens* (left), *Lactococcus lactis* NZ9000 (middle) and *Lactobacillus sake* ATCC 15521 (right). 1, W48A; 2, F51A; 3, K52A; 4, K57A; 5, K60A; 6, R65A; 7, Q56K; 8, Q61K; 9, Q56K/Q61K; 10 and 12, wild-type circularin A; 11, the control strain lacking *cirA* gene. **(B)** The SDS-PAGE protein gel to evaluate the effect of each mutant on the peptide production level. The mutants 1-9 are in the same order as the ones in the antimicrobial activity assay; WT: wild-type circularin A.

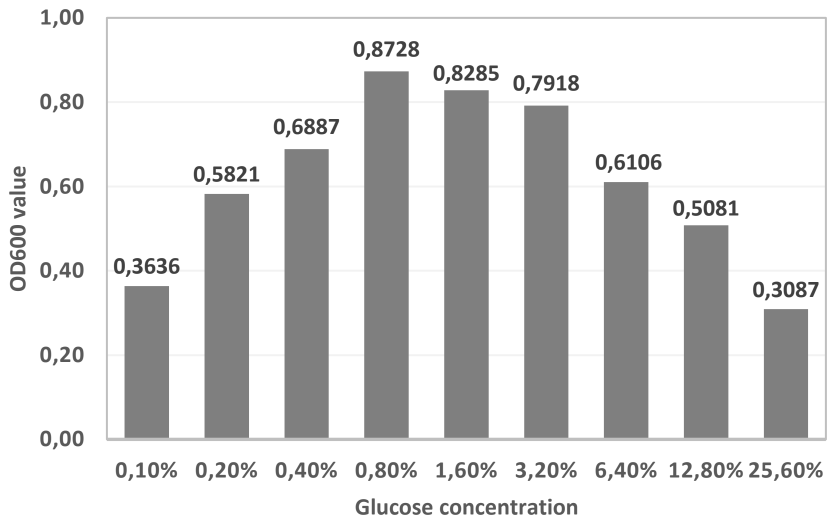

Supplementary Figure 5. The ultimate cell density of the circularin A heterologous producer (*L. lactis*) after 20 hour growth in GM17 medium with different glucose concentrations under nisin-induction.

Supplementary Table 1. Oligonucleotides used in this study

Constructs	Primers	Sequence (5'→3')
pTLR4-CirABCDE	P01	ATTATAAGGAGGCACTCAAAATGTTTTTAGTTGCAGGAGCACTAGG
	P02	CTATTTAATTTGTAATTTAGATATTATACTAGATAAAAAACCTAAGGATAC
	P03	CTAAATTACAAATTAAATAGGGAATTCAGAGGTTTGATGACTTTGACC
	P04	TTTGAGTGCCTCCTTATAATTTATTTTGTAGTTCCTTC
pTLR4-CirΔA	P05	ATGAAAAATAGCTTTTTTTATTTAATAGATATGCTAAAAATTTTATTAAAG
	P06	TAAAAAAAGCTATTTTTCATTTTGAGTGCCTCCTTATAATTTATTTTGTAGTTC
pMG-CirΔB	P07	ATAGATAGAAATATAGTCCATAAAGGAGTTG
	P08	TATATTTCTATCTATTAAATAAAAAAAGCTATTTTTCAT
pNZ-CirA	P09	ACTCACCAUGTTTTTAGTTGCAGGAGC
	P10	AAAGAAAGCUTAGTAAGCTATAGCTCTTGCCATAC
	P11	AGCTTTCTTUGAACCAAAATTAG
	P12	ATGGTGAGUGCCTCCTT
pNZ-CirB	P13	CGCGGATCCATGAAAAATAGCTTTTTTTATTTAATAGATA TGC
	P14	CCGGAATTCCTAAAACTCAAATTTTTCAACTCCTTTATG
	P15	CCGGAATTCGCTTTCTTTGAACCAAAATTAGAAAAC
	P16	CGCGGATCCTGCCTCCTTATAATTTATTTTGTAGTTC
W48A	P17	AATAGTCATUAATGTACCTGCACC
	P18	AATGACTATUGGAGCAGCAACATTCAAAGCAACAG
F51A	P17	AATAGTCATUAATGTACCTGCACC
	P19	AATGACTATUGGATGGGCAACAGCAAAAGCAACAG
K52A	P17	AATAGTCATUAATGTACCTGCACC
	P20	AATGACTATUGGATGGGCAACATTCGCAGCAACAGTT
Q56K	P21	AGCAACAGTUAAAAAATTAGCTAAGCAAAGTAT
	P22	AACTGTTGCUTTGAATGTTGCC
Q61K	P23	AGCAACAGTUCAAAAATTAGCTAAGAAAAGTAT
	P22	AACTGTTGCUTTGAATGTTGCC
Q56K/Q61K	P24	AGCAACAGTUAAAAAATTAGCTAAGAAAAGTATGGC
	P22	AACTGTTGCUTTGAATGTTGCC
K57A	P25	AGCAACAGTUCAAGCATTAGCTAAGCAAAGTATGG
	P22	AACTGTTGCUTTGAATGTTGCC
K60A	P26	AGCAACAGTUCAAAAATTAGCTGCGCAAAGTATGG
	P22	AACTGTTGCUTTGAATGTTGCC
R65A	P27	AGCAAAGTAUGGCAGCAGCTATAGCTTACTAAGC
	P28	ATACTTTGCUTAGCTAATTTTTGAACTGTTG

Supplementary Table 2. Inhibition diameters of circularin A and variants

No.	CirA variants	Inhibition diameter ($Ø_{act}$-$Ø_{col}$, mm)[a]		
		C. perfrigens	*L. Lactis*	*Lb. sake*
1	W48A	0.2	0	5.5
2	F51A	0	0	3
3	K52A	0	2	5
4	K57A	1.2	3.2	7
5	K60A	2.2	3.5	7
6	R65A	1.7	3	4
7	Q56K	1.7	2.8	8.5
8	Q61K	1.2	1.5	8
9	Q56K/Q61K	0.8	0	7
10	WT	2.5	3.6	9

[a] $Ø_{act}$: the diameter of the inhibition zone; $Ø_{col}$: the diameter of the colony of the producer strain.
